# Effects of Heat-Killed *Lactiplantibacillus plantarum* L-137 on Menopause-Related Outcomes and Gut Barrier-Related Biomarkers in Japanese Women: A Randomized, Double-Blind, Placebo-Controlled Trial

**DOI:** 10.3390/nu18213641

**Published:** 2026-10-31

**Authors:** Tsubasa Nakajima, Tatsuya Ohara, Yoshitaka Hirose, Kengo Kawasaki

**Affiliations:** Research & Development Institute, House Wellness Foods Corporation, 3-20 Imoji, Itami City 664-0011, Hyogo, Japan

**Keywords:** postbiotics, *Lactiplantibacillus plantarum* L-137, menopausal symptoms, sleep quality, gut barrier, soluble CD14, daily stress, randomized controlled trial

## Abstract

**Background/Objectives:** Menopause-related issues—such as mood disturbance, hot flashes, and sleep disturbance—are thought to be influenced not only by hormonal changes but also by everyday stress and gut barrier-related host responses. Heat-killed *Lactiplantibacillus plantarum* L-137 (HK L-137) has shown immunomodulatory and gut barrier-related effects, but clinical evidence for its effects in menopausal women is limited. We investigated whether HK L-137 intake affects menopause-related outcomes and gut barrier-related biomarkers in women reporting daily stress. **Methods:** We conducted a 12-week randomized, double-blind, placebo-controlled, parallel-group trial in healthy Japanese women aged 45–69 years who self-reported daily stress (UMIN000055687). Participants were randomly assigned to receive one tablet containing 10 mg HK L-137 or placebo daily. Mood state, menopausal symptoms, and sleep quality—as menopause-related outcomes—and gut barrier-related biomarkers were assessed at baseline and week 12. **Results:** A total of 121 participants were included, and 119 completed the study. No significant between-group differences were observed in the questionnaire scores of mood state, menopausal symptoms, or sleep quality. Changes in serum soluble CD14 (sCD14) and lactate dehydrogenase (LDH) differed significantly between groups, favoring the HK L-137 group (*p* = 0.020 and *p* = 0.010, respectively). In the prespecified subgroup with higher baseline levels of negative mood, HK L-137 significantly improved hot flashes item and sleep maintenance score, and was associated with favorable changes in sCD14, LDH, and body mass index versus placebo. No intervention-related safety concerns were identified. **Conclusions:** HK L-137 favorably affected sCD14 and LDH in the overall analysis and improved selected menopause-related outcomes in women with higher baseline levels of negative mood. These findings suggest a possible role of gut barrier-related host responses associated with microbial translocation.

## 1. Introduction

Women experience various physical and psychological changes across the life course [1,2]. Menopause is a normal stage in this; however, fluctuations and the eventual decline in female sex hormones are associated with a wide range of symptoms and conditions, including vasomotor symptoms (e.g., hot flashes), sleep disturbances, mood symptoms, fatigue, osteoporosis, and weight gain [3,4,5,6,7,8,9,10,11].

Although the prevalence of menopausal symptoms varies widely across regions, these symptoms occur globally and affect quality of life (QOL) [12,13,14,15]. Several studies have shown that menopausal symptoms—including vasomotor symptoms, such as hot flashes—are associated with reduced work productivity and an increased intention to leave employment [16,17]. In addition, a cross-sectional study of employed Japanese women reported that a greater number of menopausal symptoms was associated with poorer work performance [18]. The European Menopause and Andropause Society has issued recommendations to improve well-being and maintain work ability among menopausal women [4]. The World Health Organization has also emphasized the importance of addressing women’s health needs across the life course, including during menopause [19]. According to a report released by the Organisation for Economic Co-operation and Development (OECD) in 2025, women accounted for 58% of public servants and 46% of the total workforce across OECD member countries in 2023 [20]. Thus, research in this field is increasingly important, both for improving individual QOL and from broader societal and economic perspectives.

In addition to endocrine changes, factors such as everyday stress and inflammatory status may be related to menopausal symptoms [11,21,22,23,24,25,26]. Because menopausal symptoms may be interrelated with perceived stress, interventions targeting women who perceive everyday stress may have clinical relevance.

Hormone replacement therapy is an effective treatment option for menopausal symptoms; however, its use may be limited by eligibility considerations and contraindications, and ongoing medical supervision can be necessary [10,11]. Therefore, safe and sustainable complementary approaches are needed for a broad range of women, including those with mild-to-moderate symptoms [27]. In this context, food- and supplement-based interventions that can be readily incorporated into daily life as self-care may represent useful options.

Growing attention has been directed toward interactions between the gut environment and host responses. During the menopause, changes in the gut environment—such as alterations in the gut microbiota and increased intestinal permeability—have been suggested to be associated with enhanced translocation of microbe-derived components and increased inflammatory responses [1,28,29]. A number of pro- and postbiotics have been reported to modulate the gut microbiota and gut barrier function, and to influence immune and inflammatory responses [30,31,32,33,34]. Furthermore, some lactic acid bacteria have been reported to alleviate symptoms associated with menopause [35,36,37]. These findings suggest that such products may be promising functional food ingredients for menopausal women.

*Lactiplantibacillus plantarum* L-137 is a strain isolated from a fermented fish and rice dish that is popular in Southeast Asia [38]. Intake of heat-killed *L. plantarum* L-137 (HK L-137) has been reported to contribute to health, including improvements in health-related QOL, enhanced host defense against infections via immunomodulatory effects, improved skin condition, improvements in inflammation and lipid metabolism, and changes in the gut microbiota [39,40,41,42,43,44]. In addition, recent studies, including those using intestinal epithelial cell models, have reported findings suggestive of reduced intestinal permeability and improved barrier function [45].

However, clinical studies of HK L-137 in menopausal women remain limited, and studies exploring potential mechanisms with concomitant assessment of gut barrier-related biomarkers remain insufficient. We therefore conducted a randomized, double-blind, placebo-controlled, parallel-group trial in women aged 45–69 years who self-reported experiencing everyday stress, to evaluate the effects of 12-week HK L-137 intake on menopause-related outcomes including mood state, menopausal symptoms, and sleep quality. We also assessed gut barrier-related biomarkers to explore potential mechanisms.

## 2. Materials and Methods

### 2.1. Study Design and Ethics

This study was a 12-week, randomized, double-blind, placebo-controlled, parallel-group trial conducted between October 2024 and March 2025 at Meiwa Hospital (Tokyo, Japan) under the management of a contract research organization (CRO; Macromill Inc., Tokyo, Japan). The study was registered with the University Hospital Medical Information Network Clinical Trials Registry (UMIN-CTR; UMIN000055687; registered on 1 October 2024). The protocol was approved by the Ethics Committee of Kobuna Orthopedics Clinic (Gunma, Japan) (Approval ID: MK-2409-05; 26 September 2024) and conducted in accordance with the principles of the Declaration of Helsinki (2013). All participants provided electronic informed consent prior to enrollment. This study was designed and conducted in accordance with the Consolidated Standards of Reporting Trials (CONSORT) 2010 statement [46]. A completed CONSORT checklist, based on the CONSORT 2025 update (*BMJ* 2025;389:e081123), is provided in Supplementary Table S1.

### 2.2. Participant Enrollment

Healthy Japanese women were recruited between October and November 2024 from a panel of registered monitors managed by Macromill Inc. Women who self-reported experiencing stress in daily life were invited to participate. After completing the web-based pre-screening, candidates who met all inclusion criteria and none of the exclusion criteria proceeded to the on-site screening visit. Based on the screening results, candidates deemed eligible by the investigator were enrolled. The inclusion criteria were as follows: (1) healthy Japanese women aged 45 to 69 years at the time of providing informed consent; (2) self-reported experience of everyday stress; (3) a relatively high Total Mood Disturbance (TMD) T-score in the Profile of Mood States, Second Edition (POMS-2) Adult Short Form (Kaneko Shobo Co., Ltd., Tokyo, Japan), administered during the web-based screening; and (4) the ability to understand the study procedures and provide electronic informed consent. The exclusion criteria were as follows: (1) current medical treatment for gynecological, thyroid, autonomic nervous system, or psychiatric disorders, or planned initiation of such treatment during the study; (2) current use of prescription or over-the-counter medications that may affect menopausal symptoms, including oral contraceptives, ovulation suppressants, hormone preparations, medications for menopausal symptoms, or Kampo medicine; (3) history of oophorectomy or hysterectomy; (4) previous hormone replacement therapy; (5) current treatment for chronic diseases, including diabetes, dyslipidemia, or hypertension; (6) current treatment for, or a clinically significant history of, malignant tumors or cerebrovascular, cardiac, hepatic, renal, hematologic, endocrine, or metabolic disorders; (7) current treatment for, or a clinically significant history of, gastrointestinal diseases or gastrointestinal surgery, excluding appendectomy; (8) clinically significant abnormalities in vital signs, laboratory tests, or physical examination at screening; (9) suspected or confirmed infection with HBV, HCV, syphilis, or HIV; (10) prior adverse reactions or clinically relevant deterioration in physical condition associated with blood sampling; (11) blood donation of 400 mL within 16 weeks, 200 mL within 4 weeks, or component donation within 2 weeks prior to the screening visit, or inability to abstain from blood donation between giving informed consent and the end of the study; (12) breastfeeding, pregnant, possibly pregnant, or planning pregnancy during the study; (13) suspected mental disorder based on the Self-Rating Depression Scale score and investigator interview at web-based screening; (14) a Simplified Menopausal Index (SMI) score of ≥51 at web-based screening; (15) regular use of functional foods or dietary supplements that could not be discontinued during the study; (16) regular intake of foods or supplements containing the same active ingredient as the test tablets; (17) conditions with a risk of blood contamination in saliva samples, such as periodontal or oral disease with oral bleeding; (18) allergy to the test food or its ingredients, including milk; (19) heavy smoking (≥21 cigarettes/day), high alcohol intake (≥60 g/day of pure alcohol), or extremely irregular eating habits; (20) anticipated substantial changes in living environment, diet, or exercise habits during the study; (21) participation in another clinical trial within 4 weeks prior to screening, or planned participation in another trial during the study; or (22) deemed ineligible by the investigator.

### 2.3. Experimental Food

The study used a commercially available HK L-137 preparation (House Wellness Foods Corporation, Hyogo, Japan), containing 20% heat-killed *L. plantarum* L-137 (HK L-137) and 80% dextrin. The *L. plantarum* L-137 culture method was performed as previously reported [47].

### 2.4. Randomization and Blinding

The sponsor ensured that the active and placebo tablets were visually indistinguishable; each tablet package was labeled with an identification code. The key linking identification codes to treatment assignment (active or placebo) was provided to the allocation manager within the CRO. Within the CRO, independent personnel served as the allocation manager, study tablet manager, and statistical analysis officer to maintain blinding throughout the study. The allocation manager—who was not otherwise involved in conduction of the study—generated the allocation sequence using a computer-generated stratified block randomization method with age, baseline POMS-2 TMD T-score, and baseline SMI score as stratification factors, and assigned participants to either the HK L-137 group or the placebo group. The allocation ratio was set at 1:1. The allocation manager provided the study tablet manager with the coded allocation information required for tablet distribution, while retaining the treatment code key in a secure location. The treatment code key and allocation documents were not accessed until unblinding. The study tablet manager was responsible for the appropriate storage, accountability, and distribution of the coded study tablets and was not involved in participant assessment. The statistical analysis officer prepared the statistical analysis plan, conducted the analyses, and prepared the statistical analysis report. Unblinding was performed only after the database lock. All participants, study investigators, the study tablet manager, clinical laboratories, and the statistical analysis officer were blinded to the group allocation.

### 2.5. Intervention

Participants took one tablet/day for 12 weeks: either an active tablet containing 50 mg of the HK L-137 preparation (10 mg of HK L-137 and 40 mg of dextrin) or a placebo tablet containing 50 mg of dextrin. The study tablets were taken with water or lukewarm water without chewing. No specific time of intake was designated. Before delivery to participants, the CRO confirmed that the active and placebo tablets were visually indistinguishable.

### 2.6. Assessments

Physical measurements, vital signs, blood, urine, and saliva tests, medical interviews, and questionnaire assessments were performed at baseline and after 12 weeks of the intervention. Physical and vital sign measurements, blood, urine, and saliva sampling, and medical interviews were conducted by physicians at the hospital.

During vital sign assessment, blood pressure was measured using an upper-arm automated sphygmomanometer (HEM-7131; Omron Healthcare Co., Ltd., Kyoto, Japan) after participants rested for at least 10 min following arrival at the hospital.

For blood collection, participants were instructed to fast for at least 10 h before sampling; up to approximately 200 mL of water was permitted during the fasting period.

For urine testing, participants collected one tube of first-morning midstream urine at home on the test day and submitted the sample to the hospital.

For saliva sampling, participants were instructed to brush their teeth at home before attending the visit. Drinking, tooth brushing, smoking, and similar activities were prohibited from check-in until saliva collection. Saliva was collected by holding a cotton swab in the mouth and transferring saliva into a collection tube.

Blood and urine samples were analyzed by an external clinical laboratory (LSI Medience, Tokyo, Japan). Serum fatty acid binding protein 2 (FABP2) and soluble CD14 (sCD14) were measured using a Human FABP2/I-FABP and a Human CD14 Quantikine ELISA kit, respectively (R&D Systems, Minneapolis, MN, USA), according to the manufacturer’s instructions. Estradiol (E2), follicle-stimulating hormone (FSH), luteinizing hormone (LH), and progesterone (P4) were measured by chemiluminescent immunoassay. Lactate dehydrogenase (LDH) was measured using an IFCC-standardized UV method.

Saliva samples were analyzed by an external laboratory (Yanaihara Institute Inc., Shizuoka, Japan). Salivary cortisol was measured using the EIA method (Cortisol (Saliva) EIA/New type kit; Yanaihara Institute Inc.).

Questionnaires were completed by participants via a web-based platform. Participants also recorded daily tablet intake and subjective symptoms throughout the 12-week intervention period.

### 2.7. Questionnaires

Mood state was self-assessed by participants using the POMS-2 Adult Short Form. This questionnaire consists of 35 items rated on a 5-point Likert scale and assesses mood during the past week. The TMD T-score was calculated as an overall index of negative mood. Higher scores indicate a stronger intensity of negative mood. The primary outcome of this study was the POMS-2 TMD T-score.

Menopausal symptoms were self-assessed by participants using the SMI, a 10-item questionnaire rated on a 4-point Likert scale (hot flashes; easy sweating; cold constitution; shortness of breath or palpitations; insomnia; easy excitability or irritability; frequent worrying and depressive moods; frequent headache, dizziness, or nausea; easy fatigability; and shoulder stiffness, lumbago, or pain in the extremities) [48,49]. Higher scores indicate a stronger state.

Sleep quality was self-assessed by participants using the Oguri–Shirakawa–Azumi Sleep Inventory, MA version (OSA-MA) [50]. The OSA-MA comprises 16 items rated on a 4-point Likert scale and is completed upon waking. It yields five factor scores: Factor 1 (sleepiness on rising), Factor 2 (initiation and maintenance of sleep), Factor 3 (frequent dreaming), Factor 4 (refreshing on rising), and Factor 5 (sleep length). According to the OSA-MA manual, Factor 2 can be further summarized separately for sleep initiation and sleep maintenance. Higher scores indicate better sleep status.

Fatigue level was self-assessed by participants using a visual analog scale (VAS), ranging from 0 (no fatigue) to 100 (extreme fatigue), with higher scores indicating greater fatigue [51,52].

Stool characteristic was self-assessed by participants using the Bristol Stool Scale, a validated 7-point scale ranging from type 1 (separate hard lumps) to type 7 (watery, no solid pieces). Participants recorded their stool form once daily, based on the previous day’s bowel movements. For analysis, the average stool form score was calculated for the 1-week period prior to the intervention and at week 12 of the intervention [53,54].

### 2.8. Sample Size

The target sample size was set to be approximately similar to that of a previous study that administered heat-killed lactic acid bacteria and assessed the POMS-2 TMD T-score [55]. The target enrollment was set at 130 participants to allow for screening failures and study discontinuations. This target sample size was planned for an exploratory study and was not based on a formal sample-size calculation using a reliably estimated effect size for the primary outcome.

### 2.9. Prespecified Stratified Analyses

Participants were stratified according to baseline POMS-2 TMD T-score, using a cut-off value of 50. Participants with a TMD T-score ≥50 were classified as the high-TMD group.

### 2.10. Statistical Analysis

Statistical analyses were conducted in accordance with the prespecified statistical analysis plan. Safety outcomes were analyzed in the full analysis set (FAS), defined as all enrolled participants who consumed the study tablets at least once. Efficacy outcomes were analyzed in the per-protocol set (PPS), defined as all enrolled participants excluding those who (1) were found after study initiation not to meet the inclusion criteria or to meet any of the exclusion criteria; (2) had intervention adherence below the prespecified threshold; (3) had major violations of compliance requirements; or (4) had other major protocol violations or deviations. All analyses were performed using SPSS Statistics version 30.0 (IBM Japan Ltd., Tokyo, Japan). Values are presented as mean ± standard deviation (SD). Between-group comparisons of changes from baseline to week 12 were performed using the Mann–Whitney U test for Bristol Stool Scale score and unpaired Student’s *t*-tests for the other outcomes. All tests were two-sided, and *p* < 0.05 was considered statistically significant. No imputation was performed for missing data; analyses were conducted using available cases for each outcome. We conducted a post hoc sensitivity analysis using analysis of covariance (ANCOVA) to assess the robustness of the findings. The observed value at week 12 was modeled as the dependent variable, with treatment group, the baseline value of the corresponding outcome, and the baseline values of the stratification factors used in the stratified block randomization method included as independent variables. This analysis was not prespecified.

For safety assessment, adverse events and side effects were collected via participant self-reporting (web-based diary) and clinical evaluation by the investigator. A chi-squared test was performed between the two groups to determine the number of adverse events occurring in each group. The investigator reviewed all safety data, including physical examinations, laboratory test results, and reported symptoms, to assess the causal relationship between each event and the intervention.

## 3. Results

### 3.1. Participants and Baseline Characteristics

A total of 722 individuals who expressed interest in participating underwent web-based screening. Of these, 131 candidates who met the inclusion criteria and none of the exclusion criteria proceeded to the on-site screening visit. Based on the on-site screening results, 121 healthy participants were deemed eligible by the principal investigator and enrolled. Health status was determined by the investigator based on physical measurements, vital signs, blood and urine tests, and a medical interview. All 121 participants were randomized to either the placebo group (*n* = 61) or the HK L-137 group (*n* = 60). In the HK L-137 group, one participant did not receive the allocated intervention due to surgery for a gastric submucosal tumor before the start of study tablet intake. One additional participant in the HK L-137 group discontinued the intervention 2 weeks after initiation due to breast cancer surgery. Consequently, 119 participants completed the study (placebo group, *n* = 61; HK L-137 group, *n* = 58). Safety analyses were conducted in the FAS (placebo group, *n* = 61; HK L-137 group, *n* = 59), defined as all randomized participants who consumed at least one study tablet. Efficacy analyses were conducted in the PPS (placebo group, *n* = 61; HK L-137 group, *n* = 58). One participant in the placebo group did not attend the week 12 hospital visit for personal reasons; therefore, laboratory assessments were unavailable for this participant, but questionnaire data were collected. The participant flow is shown in Figure 1. Baseline characteristics in the FAS are shown in Table 1. There was no meaningful difference between the groups. Intervention adherence, calculated from the number of tablets returned at the 12-week visit, exceeded 98% in both groups.

### 3.2. Effects of HK L-137 on Menopause-Related Outcomes

Overall efficacy on menopause-related outcomes is summarized in Table 2. Mood state, assessed by the POMS-2 TMD T-score (the primary outcome), improved in both groups, with no significant between-group difference. Because higher SMI scores indicate more pronounced symptoms, negative changes represent improvement. The SMI total score improved numerically in both groups, but the between-group difference in change from baseline was not significant. Subjective sleep quality, assessed using OSA-MA factor scores, showed no significant between-group differences. Fatigue VAS score was significantly higher at baseline in the HK L-137 group than in the placebo group; however, fatigue improved in both groups, though no significant between-group difference was observed in the change from baseline. The post hoc ANCOVA showed no statistically significant between-group differences for any of the outcomes (Supplementary Table S3).

### 3.3. Effects of HK L-137 on Blood/Salivary Markers and Stool Characteristic

Serum biomarkers, female hormones, salivary cortisol, and stool characteristic are presented in Table 3. FABP2 and sCD14 were evaluated as gut barrier-related biomarkers. No significant difference was observed between groups for FABP2, a marker of intestinal epithelial damage. In contrast, serum sCD14, a biomarker associated with microbial translocation, showed a significant difference between groups in change from baseline to week 12 (*p* = 0.020). LDH was evaluated as an adjunct biomarker related to inflammatory status; significant differences were also observed between groups in change from baseline (*p* = 0.010). No significant differences were observed in female hormone (E2, FSH, LH, and P4) or salivary cortisol levels between groups. No significant differences were observed between groups in bowel movement assessment using the Bristol Stool Scale. In the post hoc ANCOVA, statistically significant between-group differences were observed for serum sCD14 and LDH, consistent with the prespecified analyses (Supplementary Table S4).

### 3.4. Prespecified Stratified Analysis by Baseline POMS-2 TMD T-Score

To explore the effects of HK L-137 among participants who were determined to have high levels of negative mood state based on a validated questionnaire—rather than relying on self-reported stress—we conducted a prespecified stratified analysis based on baseline POMS-2 TMD T-score cut-off of 50, in accordance with the statistical analysis plan (Table 4). T-score were standardized with a mean of 50 and an SD of 10; scores ≥50 therefore indicate higher-than-average distress.

Among participants in the high-TMD group (placebo group, *n* = 46; HK L-137 group, *n* = 42), the SMI hot flashes item increased slightly in the placebo group but decreased—indicating improvement—in the HK L-137 group (0.12 ± 1.11 vs. −0.52 ± 1.64; *p* = 0.033). The OSA-MA sleep maintenance score increased in both groups, with a larger increase in the HK L-137 group (1.07 ± 4.89 vs. 3.42 ± 5.20; *p* = 0.032). BMI increased in both groups, but the increase was smaller in the HK L-137 group (0.32 ± 0.49 vs. 0.10 ± 0.53 kg/m^2^; *p* = 0.046). Serum sCD14 increased in the placebo group but decreased in the HK L-137 group (60.54 ± 209.87 vs. −39.71 ± 200.13 ng/mL; *p* = 0.025), and serum LDH increased in the placebo group but decreased in the HK L-137 group (5.48 ± 14.66 vs. −1.83 ± 14.31 U/L; *p* = 0.020). All of these indicators showed improvement in the HK L-137 group. No significant between-group differences were observed for other outcomes, including POMS-2 measures. In the post hoc ANCOVA, statistically significant between-group differences were observed for serum sCD14 (*p* = 0.038) and LDH (*p* = 0.013), consistent with the prespecified analyses. No statistically significant between-group differences were observed for the SMI hot flashes item (*p* = 0.229), the OSA-MA sleep maintenance score (*p* = 0.083), or BMI (*p* = 0.052) (Supplementary Table S5).

### 3.5. Safety

All safety evaluations, including monitoring of adverse events, were conducted in the FAS. A total of 143 adverse events were reported in the placebo group, with 120 in the HK L-137 group. Because most events were transient and/or resolved within a short period, participants continued the trial at the investigator’s discretion. The number of participants who experienced at least one adverse event was 33/61 in the placebo group and 31/59 in the HK L-137 group, with no significant difference between groups (Supplementary Table S2). All adverse events were mild and were judged by the investigator to be unrelated to the intervention. Safety parameters (vital signs, hematology, blood biochemistry, and urinalysis) were evaluated by comparing changes from baseline to week 12. Although a statistically significant between-group difference was observed in LDH, levels remained within the reference range. Overall, these findings support the safety of continuous intake of the study tablets for 12 weeks.

## 4. Discussion

This randomized, double-blind, placebo-controlled, parallel-group trial investigated the effects of 12-week intake of HK L-137 in Japanese women aged 45–69 years who self-reported experiencing daily stress. We assessed effects on mood state, menopausal symptoms, and sleep quality, as well as gut barrier-related biomarkers to explore potential mechanisms. The intervention was well tolerated, and no safety concerns were identified during the 12-week period. In the overall analysis of the PPS, changes from baseline in the POMS-2 TMD T-score and SMI total score improved numerically in both groups, but without significant between-group differences. Similarly, no notable results were found in OSA-MA scores. In contrast, serum sCD14 and LDH increased in the placebo group but decreased in the HK L-137 group.

In the prespecified stratified analysis focusing on subjects identified as having a negative mood state—determined via a validated questionnaire rather than self-reporting—revealed significant improvements in the SMI hot flashes item and the OSA-MA sleep maintenance score in the HK L-137 group compared to the placebo group. In the same subgroup, BMI increased in both groups, but this change was smaller in the HK L-137 group. Meanwhile, serum sCD14 and LDH increased in the placebo group but decreased in the HK L-137 group. These results suggest that HK L-137 intake may be effective for individuals experiencing higher levels of negative mood.

Although no significant between-group differences were observed for the SMI and OSA-MA scores in the overall analysis, the prespecified stratified analysis showed improvements in the SMI hot flashes item, the OSA-MA sleep maintenance score, and BMI. The absence of a significant between-group difference in the overall analysis may be attributable to participant characteristics. The PPS for the overall analysis included participants with baseline TMD T-scores <50, this may have diluted the effects of the intervention.

Hot flashes are a characteristic vasomotor symptom of the menopausal transition and, together with night sweats, reflect impaired thermoregulation. Even in premenopausal women, cyclical fluctuations in sex hormones are known to influence thermoregulation, and low estradiol levels and higher progesterone-to-estradiol ratio have been associated with increased body temperature [56,57]. Vasomotor symptoms are generally considered to be related, at least in part, to the effects of estrogen deficiency on central thermoregulatory pathways [11,12,58,59]. Recent studies have also suggested involvement of inflammatory cytokines and the gut microbiota in vasomotor symptoms [60,61,62]. During the menopausal transition, increases in inflammatory cytokines (e.g., IL-6 and TNF-α) have been reported, potentially mediated by altered immune responses associated with declining estrogen levels [63,64,65]. In addition, menopause has been reported to be accompanied by gut microbiota changes and inflammation associated with increased intestinal permeability [29,66,67].

Sleep disturbance is another common complaint during the menopause. A meta-analysis investigating the relationship between menopause and sleep disorders reported that the odds of experiencing sleep problems increase by more than 1.6-fold during the menopausal transition compared with the premenopausal period [68]; hot flashes and night sweats are often linked to these sleep disturbances. Reported sleep symptoms during menopause include increases in nocturnal awakenings and wake time after sleep onset [21,69,70,71]. Multiple studies—including those from the Study of Women’s Health Across the Nation—have reported associations between vasomotor symptom severity and difficulty maintaining sleep [72,73]. Other studies have described relationships between changes in the gut microbiota, inflammatory markers, and sleep disturbances [74]. Moreover, inflammation and sleep disruption are known to be bidirectionally related: inflammation can promote sleep fragmentation, and insufficient sleep can induce inflammatory responses [75].

In midlife women, increased BMI is commonly observed [9,10]. Higher BMI has been associated with more severe hot flashes, and weight loss may contribute to the improvement of vasomotor symptoms [76]. Associations between BMI, inflammation, gut barrier function, and the gut microbiota have also been increasingly recognized [77,78,79].

Taken together, improvements in hot flashes, sleep maintenance, and prevention of BMI increase in menopausal women may result from previously reported beneficial effects of HK L-137, such as immunomodulation [39,40,41,47], anti-inflammatory effects [43,80], improvements in gut barrier-related host responses [43,45,80], and modulation of the gut microbiota [44].

The biomarker findings provide additional context for this interpretation. FABP2 and sCD14 were evaluated as gut barrier-related biomarkers. No single indicator has been established that directly demonstrates intestinal barrier failure; however, FABP2 and sCD14 are given as examples of highly relevant indicators of intestinal barrier function in clinical practice in the joint statement on Gut Health by the International Scientific Association for Probiotics and Prebiotics (ISAPP). FABP2 is considered to reflect intestinal epithelial cell injury, whereas sCD14 is regarded as a marker related to microbial translocation [29,81,82]. While FABP2 did not show a significant between-group difference, sCD14 increased in the placebo group and decreased in the HK L-137 group in both the overall and stratified analyses. This pattern suggests that HK L-137 influenced host responses related to microbial translocation rather than overt enterocyte injury. Potential mechanisms may include strengthening of tight junctions and enhancement of the mucus barrier. In an in vitro study, increased expression of the tight junction-associated protein ZO-1 and suppression of FD-4 permeability were observed in an intestinal epithelial cell line (IEC-6) following treatment with HK L-137, suggesting strengthened barrier integrity [45]. Moreover, in preclinical studies, the intake of HK L-137 has been reported increased ZO-1 expression and suppression of inflammatory cytokines under both normal diet and dextran sulfate sodium-induced colitis conditions; furthermore, a study of obese animals suggested the administration of HK L-137 suppresses weight gain through the improvement of intestinal barrier function (indicated by a decrease in plasma lipopolysaccharide-binding protein [LBP]) [80,83]. With respect to the mucus barrier, increases in the number of intestinal goblet cells in carp and increased mucus on the body surface in red sea bream have been reported after HK L-137 treatment [84,85]. Taken together, these findings provide biological plausibility that HK L-137 may influence gut barrier-related host responses, consistent with the observed changes in sCD14.

LDH also showed significant between-group differences in changes from baseline in both the overall and stratified analyses, although values remained within the reference range. LDH is a nonspecific enzyme released from multiple tissues, and may be interpreted as a supplementary marker that reflects tissue injury or inflammatory status [86]. In our study population, LDH was increased in the placebo group and decreased in the HK L-137 group. In this study, sCD14 increased in the placebo group and decreased in the HK L-137 group, and that FABP2 did not differ significantly between groups. Based on these findings, it may be hypothesized that maintenance of intestinal integrity associated with microbial translocation (e.g., via tight junctions and/or the mucus barrier) without affecting enterocyte injury may reduce downstream systemic inflammation and contribute to lower LDH. However, because LDH is not specific to any one tissue or pathway, the clinical significance of this finding should be interpreted cautiously.

Although not statistically significant, results relating to the Bristol Stool Scale showed a numerical trend toward improvement after HK L-137 intake in the overall analysis (*p* < 0.1). The ISAPP joint statement on gut health notes the utility of the Bristol Stool Scale as an indicator of bowel habits and gastrointestinal motility [81]. The scale ranges from 1 to 7, where lower scores indicate constipation, and higher scores indicate diarrhea; a score of 4 is generally considered optimal. In this study, HK L-137 intake was associated with a shift toward type 4, suggesting a potential benefit for stool form. A Japanese study reported that a Bristol Stool Scale score of 4 was associated with optimal sleep status [87]. While this finding is exploratory, it is consistent with the broader view that bowel habits, sleep quality, and gut health may be interconnected.

Changes in estradiol levels from baseline also tended to be higher in the HK L-137 group in the overall analysis (*p* < 0.1). Whereas estradiol decreased during the intervention in the placebo group, it increased in the HK L-137 group. One possible explanation is that HK L-137 may have influenced estrogen-related pathways indirectly, through effects on the gut environment. The gut microbiota has been reported to contribute to the regulation of circulating estrogen in postmenopausal women. β-glucuronidase, produced by gut bacteria, can deconjugate conjugated estrogens in the intestine, enabling their reabsorption into the bloodstream as active estrogens. As well as endogenous estrogens, β-glucuronidase can also deconjugate phytoestrogens. This type of bacterial flora—the “estrobolome”—is involved in estrogen metabolism and is attracting increasing attention in relation to its role in menopausal health [88,89,90]. Another possible pathway involves Type I interferon (IFN), which preclinical studies have reported can promote aromatase expression in adipose tissue and increase local estradiol production [91]. Meanwhile intake of HK L-137 has been shown to increase IFN-β in the peripheral blood of humans and pigs [40]; this may explain its effects on menopause in humans. In other words, HK L-137 intake may contribute to women’s health through estrobolome regulation and pathways mediated by Type I IFN. Lower estradiol levels have been associated with increased difficulty initiating and maintaining sleep [73], suggesting that the observed improvement in sleep maintenance in the present trial may be related to estrogen-related pathways. Nevertheless, these mechanistic considerations are speculative because the present study did not directly evaluate the gut microbiota or IFN-related pathways.

Previous studies have also suggested that HK L-137 intake can have effects on the gut microbiota and short-chain fatty acids [44,83]. In addition to its immunomodulatory effects, the combined influence on gut health—including on intestinal barrier function, the gut microbiota, and short-chain fatty acids—may have contributed to improvements in various outcomes. However, among these possible mechanisms, the present data most directly supports an association with gut barrier-related host responses, as reflected by the change in sCD14.

The strengths of this study include the randomized, double-blind, placebo-controlled design and the focus on women of menopausal age who self-reported daily stress. Another strength is its attempt to explore the underlying mechanisms by measuring intestinal barrier-related biomarkers in addition to clinical outcomes. Furthermore, the study provided confirmation of the safety of daily HK L-137 intake in menopausal women.

The subgroup findings suggest that HK L-137 intake may provide a greater benefit for certain menopausal-related symptoms (vasomotor and sleep-related) among women with higher baseline distress. However, although the stratified analysis was prespecified, subgroup results should be interpreted cautiously. Furthermore, as these were not the primary outcome and multiplicity was not accounted for, these findings should be considered exploratory. In a post hoc ANCOVA conducted in this subgroup, no statistically significant between-group differences were observed for the SMI hot flashes item, the OSA-MA sleep maintenance score, or BMI, in contrast to the statistically significant findings for these outcomes in the prespecified primary analysis of changes from baseline. Thus, these findings were not consistent across the analytical approaches and should be interpreted with caution. This discrepancy may reflect differences in analytical approaches, such as comparing changes from baseline in the primary analysis and adjusting for baseline values and stratification factors in the ANCOVA. In contrast, statistically significant between-group differences in serum sCD14 and LDH were observed in both analyses, indicating greater consistency across the analytical approaches. Further evaluation of effects of heterogeneity (e.g., in different countries or ethnic contexts) and replication in similar populations is warranted. In addition, the assessment of gut barrier function relied on indirect biomarkers, and further studies incorporating gut microbiota analyses are needed. Additional limitations include the use of the PPS for the efficacy analyses and the study population consisting of only Japanese women who self-reported daily stress, limiting generalizability. Furthermore, although we recorded the presence or absence of menstruation, the menopausal stage was not formally classified using menstrual history or standard staging criteria; nor did we collect data on the exact years since menopause started. Therefore, the present findings apply to women of menopausal age reporting menopausal-related symptoms, rather than a rigorously defined peri-/postmenopausal population. Because the years since menopause may influence the efficacy of HK L-137, future studies should include a detailed assessment of menopausal status and perform stratified analyses based on the time since menopause to better understand its effects.

## 5. Conclusions

In conclusion, 12 weeks of HK L-137 intake revealed no major safety concerns. In the overall analysis, significant between-group differences were observed in changes in sCD14, a gut barrier-related biomarker, and LDH, a marker reflecting tissue injury or inflammatory status. Improvements in hot flashes, sleep maintenance, BMI, sCD14, and LDH were observed in participants with high levels of psychological stress. These results suggest that HK L-137 may be beneficial for menopause-related outcomes in women with high stress levels and that its mechanism of action may be mediated through gut barrier-related host responses associated with microbial translocation. Further research is required to verify the reproducibility of these findings, including studies that comprehensively evaluate the gut environment, inflammation, and barrier function.

## Figures and Tables

**Figure 1:**
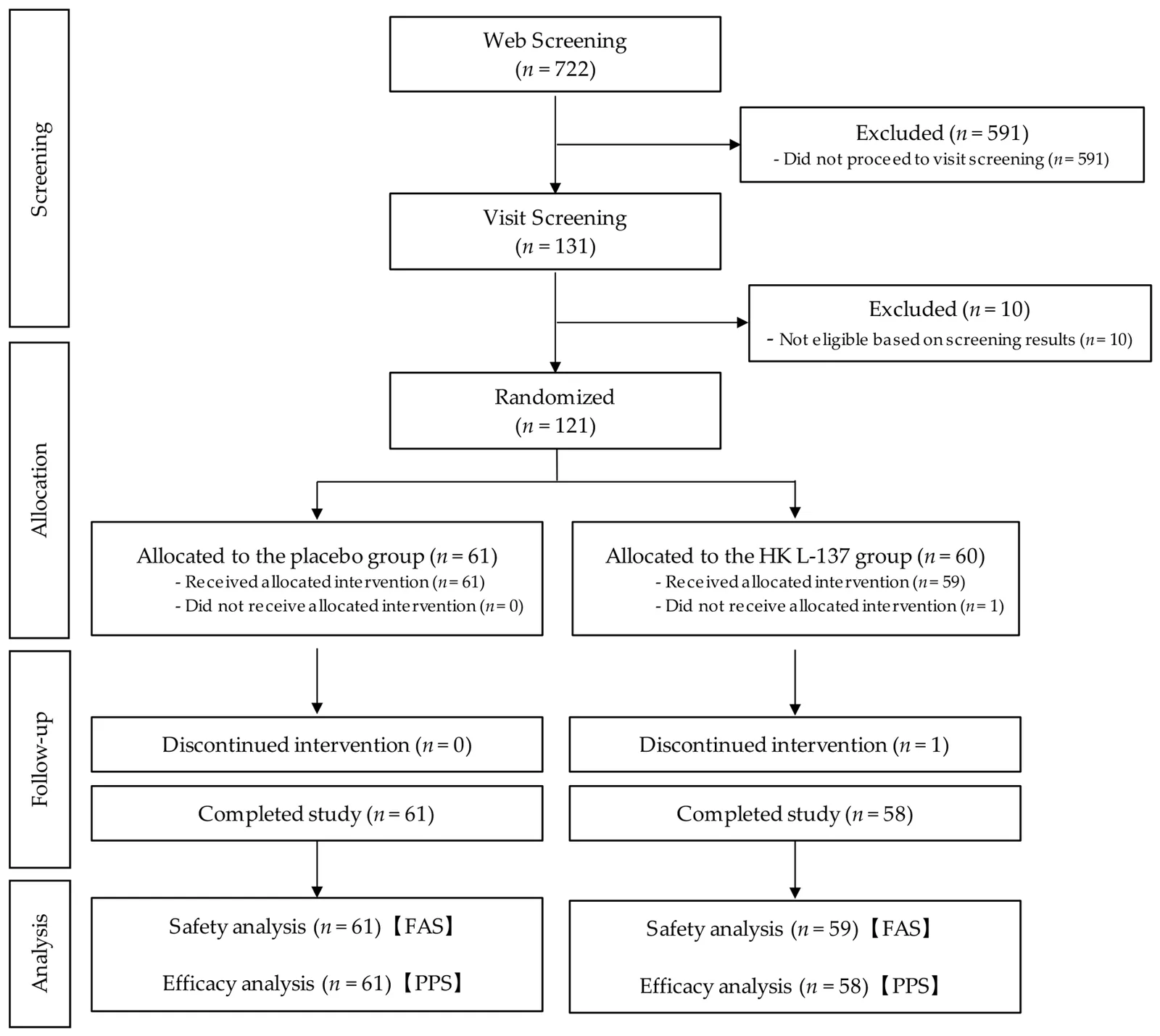
Participant flow diagram (CONSORT 2025). Of 722 women screened on the web, 131 proceeded to on-site screening and 121 were randomized to the placebo group (*n* = 61) or the HK L-137 group (*n* = 60). In the HK L-137 group, one participant did not receive the allocated intervention because of surgery for a gastric submucosal tumor before study tablet intake, and one discontinued the intervention because of breast cancer surgery 2 weeks after initiation. In the placebo group, one participant did not attend the week 12 hospital visit for laboratory assessments, although questionnaire data were available. Safety analyses were conducted in the FAS (placebo, *n* = 61; HK L-137, *n* = 59), and efficacy analyses were conducted in the PPS (placebo, *n* = 61; HK L-137, *n* = 58). Abbreviations: CONSORT, Consolidated Standards of Reporting Trials; HK L-137, heat-killed *Lactiplantibacillus plantarum* L-137; FAS, full analysis set; PPS, per-protocol set.

**Table 1:** Baseline characteristics of participants in the full analysis set (FAS).

	Placebo Group	HK L-137 Group
*n* (Menstruating/Menopause)	61 (33/28)	59 (32/27)
Age (years)	52.43 ± 5.22	52.37 ± 5.43
BMI (kg/m^2^)	21.84 ± 3.02	21.49 ± 3.00
SBP (mmHg)	108.95 ± 17.44	107.20 ± 16.18
DBP (mmHg)	74.56 ± 10.98	74.20 ± 9.93
POMS-2 TMD T-score	54.39 ± 7.43	54.49 ± 8.17
SMI total score	20.65 ± 11.53	20.80 ± 12.01

Values are presented as mean ± SD. Abbreviations: HK L-137, heat-killed *Lactiplantibacillus plantarum* L-137; BMI, body mass index; SBP, systolic blood pressure; DBP, diastolic blood pressure; POMS-2, Profile of Mood States, Second Edition; TMD, Total Mood Disturbance; SMI, Simplified Menopausal Index.

**Table 2:** Effects of HK L-137 on menopause-related outcomes in the per-protocol set (PPS).

	Baseline		Change from Baseline		
	Placebo	HK L-137	Placebo	HK L-137	*p* Value
Mood state					
POMS-2 TMD T-score	54.39 ± 7.43	54.59 ± 8.21	−3.93 ± 8.47	−1.83 ± 8.11	0.169
Menopausal symptoms					
SMI total score	20.65 ± 11.53	20.41 ± 11.73	−3.89 ± 10.82	−3.76 ± 9.16	0.947
SMI hot flashes	0.63 ± 1.17	0.82 ± 1.50	−0.06 ± 1.22	−0.47 ± 1.43	0.093
SMI easy sweating	1.53 ± 1.87	1.52 ± 1.97	−0.76 ± 1.76	−0.9 ± 1.40	0.627
SMI cold constitution	2.67 ± 3.34	1.91 ± 2.63	0.83 ± 2.96	1.13 ± 3.38	0.599
SMI shortness of breath/palpitations	0.62 ± 1.14	0.87 ± 1.69	−0.03 ± 1.50	−0.17 ± 1.20	0.578
SMI insomnia	3.86 ± 3.54	4.29 ± 3.11	−1.09 ± 3.11	−1.50 ± 3.08	0.470
SMI easy excitability or irritability	2.73 ± 2.41	2.67 ± 2.51	−0.74 ± 1.99	−0.87 ± 1.88	0.720
SMI frequent worrying and depressive moods	2.29 ± 1.84	1.79 ± 1.64	−0.67 ± 1.47	−0.24 ± 1.22	0.090
SMI frequent headache/dizziness/nausea	0.75 ± 1.10	0.75 ± 1.06	−0.22 ± 1.02	−0.06 ± 0.95	0.379
SMI easy fatigability	2.69 ± 1.55	2.61 ± 1.70	−0.85 ± 1.28	−0.56 ± 1.07	0.180
SMI shoulder stiffness, lumbago or extremities pain	2.89 ± 2.01	3.18 ± 2.04	−0.30 ± 1.43	−0.13 ± 1.66	0.555
Sleep quality					
OSA-MA Factor 1: sleepiness on rising	14.49 ± 3.48	15.22 ± 4.29	2.63 ± 5.37	1.54 ± 5.32	0.269
OSA-MA Factor 2: initiation and maintenance of sleep	17.32 ± 4.91	16.23 ± 4.40	1.52 ± 4.53	2.59 ± 4.52	0.203
OSA-MA Factor 2: sleep initiation	16.92 ± 5.21	16.29 ± 4.61	1.59 ± 5.31	2.23 ± 5.66	0.525
OSA-MA Factor 2: sleep maintenance	17.58 ± 5.45	16.19 ± 5.27	1.48 ± 4.62	2.82 ± 4.95	0.130
OSA-MA Factor 3: frequent dreaming	22.20 ± 6.06	23.09 ± 5.92	0.59 ± 4.87	−1.22 ± 5.12	0.050
OSA-MA Factor 4: refreshing on rising	15.38 ± 3.56	15.27 ± 4.27	1.83 ± 4.48	0.86 ± 4.37	0.233
OSA-MA Factor 5: sleep length	16.08 ± 4.00	16.90 ± 4.92	1.75 ± 4.83	1.36 ± 4.96	0.666
Fatigue					
Fatigue VAS score	47.30 ± 16.17	53.64 ± 18.60	−2.03 ± 22.01	−5.16 ± 23.37	0.454
Physical index					
BMI (kg/m^2^)	21.81 ± 3.03	21.51 ± 3.02	0.21 ± 0.52	0.10 ± 0.60	0.281

Values are presented as mean ± SD. Data were available for all questionnaire-based outcomes in 61 participants in the placebo group and 58 participants in the HK L-137 group; for BMI, data were available for 60 and 58 participants, respectively. The *p* values indicate between-group comparisons of changes from baseline to week 12 using unpaired Student’s *t*-tests. Higher scores indicate worse status for POMS-2 TMD, SMI, and fatigue VAS, and better status for OSA-MA. Abbreviations: HK L-137, heat-killed *Lactiplantibacillus plantarum* L-137; POMS-2, Profile of Mood States, Second Edition; TMD, Total Mood Disturbance; SMI, Simplified Menopausal Index; OSA-MA, Oguri–Shirakawa–Azumi Sleep Inventory, MA version; VAS, visual analog scale; BMI, body mass index.

**Table 3:** Effects of HK L-137 on serum biomarkers, female hormones, salivary cortisol, and stool characteristic in the per-protocol set (PPS).

	Baseline		Change from Baseline		
	Placebo	HK L-137	Placebo	HK L-137	*p* Value
Serum biomarkers					
FABP2 (pg/mL)	1449.65 ± 653.39	1591.40 ± 775.88	158.32 ± 582.49	37.22 ± 908.37	0.389
sCD14 (ng/mL)	1401.65 ± 256.81	1430.12 ± 261.05	51.13 ± 219.73	−36.67 ± 183.25	0.020
LDH (U/L)	176.67 ± 28.03	178.88 ± 27.38	4.95 ± 15.19	−2.14 ± 14.01	0.010
Female hormones					
E2 (pg/mL)	87.67 ± 147.25	51.43 ± 79.07	−16.40 ± 137.50	28.71 ± 157.18	0.099
FSH (ng/mL)	46.10 ± 33.47	49.79 ± 36.02	2.65 ± 16.17	1.45 ± 23.05	0.744
LH (mIU/mL)	19.81 ± 14.02	20.10 ± 13.16	−2.24 ± 8.81	−1.35 ± 11.97	0.647
P4 (mIU/mL)	1.63 ± 4.53	1.66 ± 3.74	−0.74 ± 5.26	−0.27 ± 4.83	0.614
Salivary biomarker					
cortisol (μg/dL)	0.21 ± 0.11	0.19 ± 0.11	−0.00 ± 0.14	0.00 ± 0.17	0.895
Stool characteristic					
Bristol Stool Scale score	3.70 ± 1.03	3.42 ± 0.99	−0.04 ± 0.74	0.28 ± 0.83	0.068

Values are presented as mean ± SD. Data were available for blood- and saliva-based outcomes in 60 participants in the placebo group and 58 participants in the HK L-137 group; for the Bristol Stool Scale score, data were available for 61 and 58 participants, respectively. The p values indicate between-group comparisons of changes from baseline to week 12. Unpaired Student’s *t*-tests were used for all outcomes except the Bristol Stool Scale score, for which the Mann–Whitney U test was used. Abbreviations: HK L-137, heat-killed *Lactiplantibacillus plantarum* L-137; FABP2, fatty acid-binding protein 2; sCD14, soluble CD14; LDH, lactate dehydrogenase; E2, estradiol; FSH, follicle-stimulating hormone; LH, luteinizing hormone; P4, progesterone.

**Table 4:** Prespecified stratified analysis in participants with baseline POMS-2 TMD T-score ≥ 50.

	Baseline		Change from Baseline		
	Placebo	HK L-137	Placebo	HK L-137	*p* Value
*n*	46	42	46	42	
Mood state					
POMS-2 TMD T-score	57.20 ± 6.57	57.81 ± 7.29	−5.39 ± 8.96	−3.10 ± 8.72	0.227
Menopausal symptoms					
SMI total score	20.53 ± 11.59	21.95 ± 12.44	−3.12 ± 10.95	−3.34 ± 9.43	0.917
SMI hot flashes	0.42 ± 0.83	0.92 ± 1.66	0.12 ± 1.11	−0.52 ± 1.64	0.033
SMI easy sweating	1.22 ± 1.53	1.68 ± 2.08	−0.52 ± 1.51	−1.00 ± 1.56	0.150
SMI cold constitution	2.64 ± 3.53	2.00 ± 2.79	0.83 ± 2.94	1.75 ± 3.62	0.194
SMI shortness of breath/palpitations	0.65 ± 1.13	1.01 ± 1.84	0.12 ± 1.55	−0.24 ± 1.26	0.227
SMI insomnia	3.71 ± 3.73	4.29 ± 3.08	−0.88 ± 3.04	−1.13 ± 2.83	0.697
SMI easy excitability or irritability	2.99 ± 2.34	3.22 ± 2.65	−0.77 ± 2.17	−1.12 ± 2.05	0.445
SMI frequent worrying and depressive moods	2.59 ± 1.91	1.89 ± 1.68	−0.71 ± 1.60	−0.15 ± 1.34	0.082
SMI frequent headache/dizziness/nausea	0.73 ± 1.13	0.84 ± 1.14	−0.12 ± 1.02	−0.17 ± 0.80	0.799
SMI easy fatigability	2.68 ± 1.69	2.77 ± 1.80	−0.85 ± 1.34	−0.52 ± 1.00	0.189
SMI shoulder stiffness, lumbago or extremities pain	2.90 ± 1.96	3.33 ± 2.06	−0.32 ± 1.49	−0.23 ± 1.34	0.770
Sleep quality					
OSA-MA Factor 1: sleepiness on rising	14.48 ± 3.54	15.00 ± 4.42	3.14 ± 5.62	1.91 ± 5.56	0.305
OSA-MA Factor 2: initiation and maintenance of sleep	17.69 ± 5.07	16.25 ± 4.26	1.30 ± 4.82	3.20 ± 4.61	0.063
OSA-MA Factor 2: sleep initiation	17.46 ± 5.20	16.33 ± 4.48	1.65 ± 5.56	2.87 ± 5.91	0.324
OSA-MA Factor 2: sleep maintenance	17.85 ± 5.69	16.20 ± 5.40	1.07 ± 4.89	3.42 ± 5.20	0.032
OSA-MA Factor 3: frequent dreaming	21.90 ± 6.42	23.56 ± 5.72	0.48 ± 5.09	−0.68 ± 5.05	0.286
OSA-MA Factor 4: refreshing on rising	15.50 ± 3.82	14.96 ± 4.57	2.16 ± 4.56	1.28 ± 4.31	0.353
OSA-MA Factor 5: sleep length	16.09 ± 4.00	16.91 ± 5.47	2.05 ± 4.99	1.08 ± 5.24	0.379
Fatigue					
Fatigue VAS score	47.09 ± 15.36	54.48 ± 18.30	−2.61 ± 22.32	−6.29 ± 23.40	0.453
Physical index					
BMI (kg/m^2^)	21.88 ± 3.18	21.49 ± 2.95	0.32 ± 0.49	0.10 ± 0.53	0.046
Serum biomarkers					
FABP2 (pg/mL)	1437.24 ± 634.48	1651.05 ± 824.10	145.02 ± 498.59	23.21 ± 747.34	0.367
sCD14 (ng/mL)	1404.61 ± 227.29	1449.17 ± 253.93	60.54 ± 209.87	−39.71 ± 200.13	0.025
LDH (U/L)	179.11 ± 29.83	181.40 ± 29.07	5.48 ± 14.66	−1.83 ± 14.31	0.020

Values are presented as mean ± SD. The p values indicate between-group comparisons of changes from baseline to week 12 using unpaired Student’s *t*-tests. Abbreviations: HK L-137, heat-killed *Lactiplantibacillus plantarum* L-137; POMS-2, Profile of Mood States, Second Edition; TMD, Total Mood Disturbance; SMI, Simplified Menopausal Index; OSA-MA, Oguri–Shirakawa–Azumi Sleep Inventory, MA version; VAS, visual analog scale; BMI, body mass index; FABP2, fatty acid-binding protein 2; sCD14, soluble CD14; LDH, lactate dehydrogenase.

## Data Availability

The original contributions presented in this study are included in the article. Further inquiries can be directed to the corresponding authors.

## Supplementary Materials

The following supporting information can be downloaded at https://www.mdpi.com/article/10.3390/nu18213641/s1, Table S1: CONSORT 2025 checklist of information to include when reporting a randomized trial. Table S2: Summary of adverse events in the full analysis set (FAS). Table S3: Post hoc analysis of menopause-related outcomes at week 12 by ANCOVA. Table S4: Post hoc analysis of serum biomarkers, female hormones, salivary cortisol, and stool characteristic at week 12 by ANCOVA. Table S5: Post hoc analysis at week 12 by ANCOVA in participants with baseline POMS-2 TMD T-score ≥ 50. The original article introducing the CONSORT 2025 checklist was published in *The BMJ* (2025;389:e081123).
