# Supplementary material for: Effects of Heat-Killed Lactiplantibacillus plantarum L-137 on Menopause-Related Outcomes and Gut Barrier-Related Biomarkers in Japanese Women: A Randomized, Double-Blind, Placebo-Controlled Trial
Source: Nutrients. 2026 Oct 31;18(21):3641. doi: 10.3390/nu18213641 (PMC13764889; doi:10.3390/nu18213641)
Supplement: Supplementary file 1 [file nutrients-18-03641-s001.zip › nutrients-4514815-supplementary.pdf]

Supplementary Materials

Table S1 CONSORT 2025 checklist of information to include when reporting a randomized trial*

Section/Topic	Item No	Checklist item	Reported on page No
**Title and abstract**			
Title and structured abstract	1a	Identification as a randomised trial	Title
	1b	Structured summary of the trial design, methods, results, and conclusions	Abstract
**Open science**			
Trial registration	2	Name of trial registry, identifying number (with URL) and date of registration	Materials & Methods – Study Design and Ethics
Protocol and statistical analysis plan	3	Where the trial protocol and statistical analysis plan can be accessed	Materials & Methods
Data sharing	4	Where and how the individual de-identified participant data (including data dictionary), statistical code and any other materials can be accessed	Data availability
Funding and conflicts of interest	5a	Sources of funding and other support (eg, supply of drugs), and role of funders in the design, conduct, analysis and reporting of the trial	Funding
	5b	Financial and other conflicts of interest of the manuscript authors	Conflict of Interest
**Introduction**			
Background and rationale	6	Scientific background and rationale	Introduction
Objectives	7	Specific objectives related to benefits and harms	Introduction
**Methods**			
Patient and public involvement	8	Details of patient or public involvement in the design, conduct and reporting of the trial	N/A
Trial design	9	Description of trial design including type of trial (eg, parallel group, crossover), allocation ratio, and framework (eg, superiority, equivalence, non-inferiority, exploratory)	Materials & Methods – Study Design and Ethics; Randomization and Blinding
Changes to trial protocol	10	Important changes to the trial after it commenced including any outcomes or analyses that were not prespecified, with reason	N/A
Trial setting	11	Settings (eg, community, hospital) and locations (eg, countries, sites) where the trial was conducted	Materials & Methods – Study Design and Ethics
Eligibility criteria	12a	Eligibility criteria for participants	Materials & Methods – Participant Enrollment
	12b	If applicable, eligibility criteria for sites and for individuals delivering the interventions (eg, surgeons, physiotherapists)	Materials & Methods – Study Design and Ethics
Intervention and comparator	13	Intervention and comparator with sufficient details to allow replication. If relevant, where additional materials describing the intervention and comparator (eg, intervention manual) can be accessed	Materials & Methods – Experimental Food (LP20);

			Intervention (intake of study tablets)
Outcomes	14	Prespecified primary and secondary outcomes, including the specific measurement variable (eg, systolic blood pressure), analysis metric (eg, change from baseline, final value, time to event), method of aggregation (eg, median, proportion), and time point for each outcome	Materials & Methods – Assessments; Questionnaires; Statistical Analysis
Harms	15	How harms were defined and assessed (eg, systematically, non-systematically)	Materials & Methods – Statistical Analysis; Assessments
Sample size	16a	How sample size was determined, including all assumptions supporting the sample size calculation	Materials & Methods – Sample Size
	16b	Explanation of any interim analyses and stopping guidelines	N/A
Randomisation:			
Sequence generation	17a	Who generated the random allocation sequence and the method used	Materials & Methods – Randomization and Blinding
	17b	Type of randomisation and details of any restriction (eg, stratification, blocking and block size)	Materials & Methods – Randomization and Blinding
Allocation concealment mechanism	18	Mechanism used to implement the random allocation sequence (eg, central computer/telephone; sequentially numbered, opaque, sealed containers), describing any steps to conceal the sequence until interventions were assigned	Materials & Methods – Randomization and Blinding
Implementation	19	Whether the personnel who enrolled and those who assigned participants to the interventions had access to the random allocation sequence	Materials & Methods – Randomization and Blinding
Blinding	20a	Who was blinded after assignment to interventions (eg, participants, care providers, outcome assessors, data analysts)	Materials & Methods – Randomization and Blinding
	20b	If blinded, how blinding was achieved and description of the similarity of interventions	Materials & Methods – Randomization and Blinding
Statistical methods	21a	Statistical methods used to compare groups for primary and secondary outcomes, including harms	Materials & Methods – Statistical Analysis
	21b	Definition of who is included in each analysis (eg, all randomised participants), and in which group	Materials & Methods – Statistical Analysis
	21c	How missing data were handled in the analysis	Results – Participants and Baseline Characteristics
	21d	Methods for any additional analyses (eg, subgroup and sensitivity analyses), distinguishing prespecified from post hoc	Materials & Methods – Prespecified Stratified Analyses; Statistical Analysis
**Results**			
Participant flow, including flow diagram	22a	For each group, the numbers of participants who were randomly assigned, received intended intervention, and were analysed for the primary outcome	Results – Participants and Baseline Characteristics (Fig.1)
	22b	For each group, losses and exclusions after randomisation, together with reasons	Results – Participants and Baseline Characteristics (Fig.1)
Recruitment	23a	Dates defining the periods of recruitment and follow-up for outcomes of benefits and harms	Materials & Methods – Study Design and Ethics; Participant Enrollment
	23b	If relevant, why the trial ended or was stopped	N/A

Intervention and comparator delivery	24a	Intervention and comparator as they were actually administered (eg, where appropriate, who delivered the intervention/comparator, how participants adhered, whether they were delivered as intended (fidelity))	Results – Participants and Baseline Characteristics
	24b	Concomitant care received during the trial for each group	N/A
Baseline data	25	A table showing baseline demographic and clinical characteristics for each group	Results – Table 1
Numbers analysed, outcomes and estimation	26	For each primary and secondary outcome, by group:<br>● the number of participants included in the analysis<br>● the number of participants with available data at the outcome time point<br>● result for each group, and the estimated effect size and its precision (such as 95% confidence interval)<br>● for binary outcomes, presentation of both absolute and relative effect size	Materials & Methods – Statistical Analysis; Results – Tables, Fig.1
Harms	27	All harms or unintended events in each group	Results – Safety
Ancillary analyses	28	Any other analyses performed, including subgroup and sensitivity analyses, distinguishing pre-specified from post hoc	Results – Prespecified Stratified Analysis by Baseline POMS-2 TMD T-score
**Discussion**			
Interpretation	29	Interpretation consistent with results, balancing benefits and harms, and considering other relevant evidence	Discussion
Limitations	30	Trial limitations, addressing sources of potential bias, imprecision, generalisability, and, if relevant, multiplicity of analyses	Discussion – Strengths and limitations

* Citation: Hopewell S, Chan AW, Collins GS, Hróbjartsson A, Moher D, Schulz KF, et al. CONSORT 2025 Statement: updated guideline for reporting randomised trials. BMJ. 2025; 388:e081123. https://dx.doi.org/10.1136/bmj-2024-081123

© 2025 Hopewell et al. This is an Open Access article distributed under the terms of the Creative Commons Attribution License (https://creativecommons.org/licenses/by/4.0/), which permits unrestricted use, distribution, and reproduction in any medium, provided the original work is properly cited.

We strongly recommend reading this statement in conjunction with the CONSORT 2025 Explanation and Elaboration and/or the CONSORT 2025 Expanded Checklist for important clarifications on all the items. We also recommend reading relevant CONSORT extensions. See www.consort-spirit.org.

Table S2 Summary of adverse events in the full analysis set (FAS)

	Placebo group	HK L-137 group	p value
n	61	59	-
Total number of adverse events	143	120	-
Participants with ≥1 adverse event, n (%)	33 (54.1)	31 (52.5)	0.864

Values are presented as number or number (%). The p value was calculated using the chi-squared test. All adverse events were mild and were judged unrelated to the study tablets. Abbreviations: HK L-137, heat-killed *Lactiplantibacillus plantarum* L-137.

Table S3 Post hoc analysis of menopause-related outcomes at week 12 by ANCOVA

	Adjusted mean (95% CI)		Adjusted mean difference (95% CI)	p value
	Placebo	HK L-137		
Mood state				
POMS-2 TMD T-score	50.50 (48.65, 52.36)	52.71 (50.81, 54.61)	2.21 (-0.45, 4.86)	0.103
Menopausal symptoms				
SMI total score	16.67 (14.16, 19.19)	16.74 (14.16, 19.32)	0.07 (-3.54, 3.67)	0.970
SMI hot flashes	0.59 (0.32, 0.86)	0.33 (0.05, 0.60)	-0.26 (-0.65, 0.12)	0.172
SMI easy sweating	0.77 (0.48, 1.06)	0.63 (0.33, 0.93)	-0.14 (-0.56, 0.28)	0.509
SMI cold constitution	3.25 (2.46, 4.04)	3.29 (2.49, 4.10)	0.04 (-1.10, 1.18)	0.943
SMI shortness of breath/palpitations	0.66 (0.35, 0.97)	0.63 (0.31, 0.94)	-0.03 (-0.48, 0.41)	0.878
SMI insomnia	2.84 (2.18, 3.49)	2.72 (2.05, 3.40)	-0.11 (-1.06, 0.83)	0.811
SMI easy excitability or irritability	1.97 (1.53, 2.42)	1.83 (1.37, 2.28)	-0.15 (-0.78, 0.49)	0.650
SMI frequent worrying and depressive moods	1.46 (1.14, 1.78)	1.72 (1.39, 2.05)	0.27 (-0.20, 0.73)	0.261
SMI frequent headache/dizziness/nausea	0.52 (0.30, 0.75)	0.69 (0.46, 0.92)	0.17 (-0.15, 0.49)	0.306
SMI easy fatigability	1.81 (1.53, 2.09)	2.07 (1.79, 2.36)	0.26 (-0.13, 0.66)	0.192
SMI shoulder stiffness, lumbago or extremities pain	2.70 (2.32, 3.08)	2.94 (2.55, 3.32)	0.24 (-0.30, 0.78)	0.384
Sleep quality				
OSA-MA Factor 1: sleepiness on rising	17.47 (16.11, 18.83)	16.39 (15.00, 17.79)	-1.08 (-3.03, 0.88)	0.278
OSA-MA Factor 2: initiation and maintenance of sleep	18.49 (17.39, 19.59)	19.18 (18.05, 20.31)	0.69 (-0.90, 2.28)	0.389
OSA-MA Factor 2: sleep initiation	18.32 (16.97, 19.66)	18.72 (17.34, 20.10)	0.40 (-1.53, 2.33)	0.682
OSA-MA Factor 2: sleep maintenance	18.61 (17.47, 19.76)	19.48 (18.30, 20.65)	0.86 (-0.79, 2.51)	0.301
OSA-MA Factor 3: frequent dreaming	23.13 (21.92, 24.34)	21.52 (20.28, 22.76)	-1.61 (-3.34, 0.13)	0.069
OSA-MA Factor 4: refreshing on rising	17.17 (16.06, 18.29)	16.17 (15.02, 17.31)	-1.01 (-2.61, 0.59)	0.215
OSA-MA Factor 5: sleep length	18.16 (16.95, 19.37)	17.91 (16.67, 19.15)	-0.25 (-1.99, 1.49)	0.777
Fatigue				
Fatigue VAS score	45.33 (41.35, 49.30)	48.41 (44.34, 52.49)	3.08 (-2.66, 8.83)	0.290
Physical index				
BMI (kg/m^2)	21.89 (21.75, 22.02)	21.75 (21.61, 21.89)	-0.14 (-0.34, 0.06)	0.162

This analysis was not prespecified in the statistical analysis plan. The adjusted mean was adjusted for age, baseline POMS-2 TMD T-score, baseline SMI total score, and the baseline value corresponding to each outcome. Results are given as adjusted means and 95 % Confidence Interval. The adjusted mean difference represents the HK L-137 group minus the placebo group. Data were available for all questionnaire-based outcomes in 61 participants in the placebo group and 58 participants in the HK L-137 group; for BMI, data were available for 60 and 58 participants, respectively. Higher scores indicate worse status for POMS-2 TMD, SMI, and fatigue VAS, and better status for OSA-MA. Abbreviations: HK L-137, heat-killed *Lactiplantibacillus plantarum* L-137; POMS-2, Profile of Mood States, Second Edition; TMD, Total Mood Disturbance; SMI, Simplified Menopausal Index; OSA-MA, Oguri–Shirakawa–Azumi Sleep Inventory, MA version; VAS, visual analogue scale; BMI, body mass index.

Table S4 Post hoc analysis of serum biomarkers, female hormones, salivary cortisol, and stool characteristic at week 12 by ANCOVA

	Adjusted mean (95% CI)		Adjusted mean difference (95% CI)	p value
	Placebo	HK L-137		
Serum biomarkers				
FABP2 (pg/mL)	1666.16 (1473.82, 1858.50)	1568.42 (1372.77, 1764.07)	-97.74 (-372.85, 177.37)	0.483
sCD14 (ng/mL)	1461.14 (1415.82, 1506.45)	1384.81 (1338.72, 1430.90)	-76.33 (-141.02, -11.64)	0.021
LDH (U/L)	182.67 (178.95, 186.38)	175.65 (171.88, 179.43)	-7.01 (-12.31, -1.72)	0.010
Female hormones				
E2 (pg/mL)	63.47 (28.89, 98.05)	88.2 (53.02, 123.39)	24.74 (-24.95, 74.42)	0.326
FSH (ng/mL)	50.27 (45.27, 55.28)	49.65 (44.56, 54.74)	-0.62 (-7.77, 6.53)	0.864
LH (mIU/mL)	17.62 (15.41, 19.83)	18.70 (16.45, 20.95)	1.07 (-2.08, 4.23)	0.501
P4 (mIU/mL)	0.90 (-0.05, 1.85)	1.38 (0.41, 2.34)	0.47 (-0.88, 1.83)	0.491
Salivary biomarker				
cortisol (μg/dL)	0.21 (0.17, 0.24)	0.20 (0.16, 0.23)	-0.01 (-0.06, 0.04)	0.648
Stool characteristic				
Bristol Stool Scale score	3.57 (3.39, 3.75)	3.80 (3.61, 3.98)	0.22 (-0.03, 0.48)	0.083

This analysis was not prespecified in the statistical analysis plan. The adjusted mean was adjusted for age, baseline POMS-2 TMD T-score, baseline SMI total score, and the baseline value corresponding to each outcome. Results are given as adjusted means and 95 % Confidence Interval. The adjusted mean difference represents the HK L-137 group minus the placebo group. Data were available for blood- and saliva-based outcomes in 60 participants in the placebo group and 58 participants in the HK L-137 group; for the Bristol Stool Scale score, data were available for 61 and 58 participants, respectively. Abbreviations: HK L-137, heat-killed *Lactiplantibacillus plantarum* L-137; FABP2, fatty acid-binding protein 2; sCD14, soluble CD14; LDH, lactate dehydrogenase; E2, estradiol; FSH, follicle-stimulating hormone; LH, luteinizing hormone; P4, progesterone.

Table S5 Post hoc analysis at week 12 by ANCOVA in participants with baseline POMS-2 TMD T-score ≥ 50

	Adjusted mean (95% CI)		Adjusted mean difference (95% CI)	p value
	Placebo	HK L-137		
Mood state				
POMS-2 TMD T-score	51.81 (49.44, 54.19)	54.51 (52.02, 57.00)	2.70 (-0.75, 6.15)	0.123
Menopausal symptoms				
SMI total score	17.97 (14.95, 20.98)	18.00 (14.84, 21.16)	0.04 (-4.34, 4.41)	0.987
SMI hot flashes	0.62 (0.27, 0.96)	0.31 (-0.05, 0.67)	-0.31 (-0.81, 0.20)	0.229
SMI easy sweating	0.79 (0.43, 1.14)	0.57 (0.20, 0.95)	-0.21 (-0.73, 0.31)	0.420
SMI cold constitution	3.27 (2.32, 4.22)	3.97 (2.97, 4.96)	0.70 (-0.69, 2.09)	0.319
SMI shortness of breath/palpitations	0.88 (0.51, 1.26)	0.64 (0.25, 1.03)	-0.25 (-0.79, 0.29)	0.366
SMI insomnia	3.01 (2.25, 3.77)	2.96 (2.16, 3.75)	-0.05 (-1.16, 1.05)	0.922
SMI easy excitability or irritability	2.30 (1.74, 2.86)	2.03 (1.44, 2.62)	-0.27 (-1.08, 0.55)	0.513
SMI frequent worrying and depressive moods	1.66 (1.25, 2.07)	1.97 (1.54, 2.40)	0.31 (-0.29, 0.92)	0.309
SMI frequent headache/dizziness/nausea	0.63 (0.40, 0.87)	0.63 (0.38, 0.88)	0.00 (-0.34, 0.34)	0.995
SMI easy fatigability	1.87 (1.54, 2.20)	2.21 (1.86, 2.55)	0.34 (-0.14, 0.81)	0.162
SMI shoulder stiffness, lumbago or extremities pain	2.76 (2.34, 3.17)	2.91 (2.47, 3.34)	0.15 (-0.45, 0.76)	0.618
Sleep quality				
OSA-MA Factor 1: sleepiness on rising	17.80 (16.14, 19.45)	16.72 (14.98, 18.45)	-1.08 (-3.49, 1.33)	0.376
OSA-MA Factor 2: initiation and maintenance of sleep	18.49 (17.14, 19.85)	20.00 (18.58, 21.42)	1.51 (-0.47, 3.48)	0.134
OSA-MA Factor 2: sleep initiation	18.70 (17.07, 20.33)	19.64 (17.93, 21.35)	0.94 (-1.43, 3.31)	0.432
OSA-MA Factor 2: sleep maintenance	18.38 (16.95, 19.80)	20.21 (18.72, 21.70)	1.83 (-0.25, 3.90)	0.083
OSA-MA Factor 3: frequent dreaming	23.01 (21.59, 24.44)	22.20 (20.70, 23.69)	-0.82 (-2.90, 1.27)	0.438
OSA-MA Factor 4: refreshing on rising	17.43 (16.13, 18.74)	16.48 (15.12, 17.85)	-0.95 (-2.85, 0.95)	0.322
OSA-MA Factor 5: sleep length	18.41 (16.95, 19.87)	17.69 (16.16, 19.22)	-0.72 (-2.85, 1.41)	0.502
Fatigue				
Fatigue VAS score	44.53 (39.84, 49.22)	48.13 (43.22, 53.04)	3.59 (-3.28, 10.47)	0.301
Physical index				
BMI (kg/m^2)	22.01 (21.86, 22.15)	21.79 (21.64, 21.95)	-0.21 (-0.43, 0.00)	0.052
Serum biomarkers				
FABP2 (pg/mL)	1662.48 (1491.89, 1833.08)	1586.40 (1407.74, 1765.05)	-76.09 (-324.92, 172.74)	0.545
sCD14 (ng/mL)	1476.20 (1425.02, 1527.38)	1397.35 (1343.78, 1450.93)	-78.84 (-153.19, -4.50)	0.038
LDH (U/Ｌ)	185.93 (181.69, 190.17)	178.10 (173.66, 182.54)	-7.83 (-13.98, -1.68)	0.013

This analysis was not prespecified in the statistical analysis plan. The adjusted mean was adjusted for age, baseline POMS-2 TMD T-score, baseline SMI total score, and the baseline value corresponding to each outcome. Results are given as adjusted means and 95 % Confidence Interval. The adjusted mean difference represents the HK L-137 group minus the placebo group. Data were available in 46 participants in the placebo group and 42 participants in the HK L-137 group. Abbreviations: HK L-137, heat-killed *Lactiplantibacillus plantarum* L-137; POMS-2, Profile of Mood States, Second Edition; TMD, Total Mood Disturbance; SMI, Simplified Menopausal Index; OSA-MA, Oguri–Shirakawa–Azumi Sleep Inventory, MA version; VAS, visual analogue scale; BMI, body mass index; FABP2, fatty acid-binding protein 2; sCD14, soluble CD14; LDH, lactate dehydrogenase.
